# Polycystin-1 Regulates Actomyosin Contraction and the Cellular Response to Extracellular Stiffness

**DOI:** 10.1038/s41598-019-53061-0

**Published:** 2019-11-12

**Authors:** Elisa Agnese Nigro, Gianfranco Distefano, Marco Chiaravalli, Vittoria Matafora, Maddalena Castelli, Angela Pesenti Gritti, Angela Bachi, Alessandra Boletta

**Affiliations:** 1 IRCCS San Raffaele Scientific Institute, Molecular Basis of Cystic Kidney Disorders, Division of Genetics and Cell Biology, Milan, Italy; 2 IFOM-FIRC Institute of Molecular Oncology, Milan, Italy

**Keywords:** Mechanotransduction, Mechanisms of disease, Polycystic kidney disease

## Abstract

Polycystin-1 (PC-1) and 2 (PC-2) are the products of the *PKD1* and *PKD2* genes, which are mutated in Autosomal Dominant Polycystic Kidney Disease (ADPKD). They form a receptor/channel complex that has been suggested to function as a mechanosensor, possibly activated by ciliary bending in the renal tubule, and resulting in calcium influx. This model has recently been challenged, leaving the question as to which mechanical stimuli activate the polycystins still open. Here, we used a SILAC/Mass-Spec approach to identify intracellular binding partners of tagged-endogenous PC-1 whereby we detected a class of interactors mediating regulation of cellular actomyosin contraction. Accordingly, using gain and loss-of-function cellular systems we found that PC-1 negatively regulates cellular contraction and YAP activation in response to extracellular stiffness. Thus, PC-1 enables cells to sense the rigidity of the extracellular milieu and to respond appropriately. Of note, in an orthologous murine model of PKD we found evidence of increased actomyosin contraction, leading to enhanced YAP nuclear translocation and transcriptional activity. Finally, we show that inhibition of ROCK-dependent actomyosin contraction by Fasudil reversed YAP activation and significantly improved disease progression, in line with recent studies. Our data suggest a possible direct role of PC-1 as a mechanosensor of extracellular stiffness.

## Introduction

Autosomal Dominant Polycystic Kidney Disease (ADPKD) is a relatively common monogenic disorder, affecting approximately 1:2000 individuals^1,2^. The disease is due to mutation in either the *PKD1* or the *PKD2* genes, encoding for two large membrane proteins, Polycystin-1 (PC-1) and Polycystin-2 (PC-2), respectively. PC-1 is a transmembrane protein with a long extracellular domain, 11 transmembrane domains and a short intracellular C-tail^2–4^. PC-1 C-terminal interacts through a coiled-coil domain with the C-terminal of PC-2, a cation channel of the TRPP family, to form the PC-1/PC-2 complex, likely with an intrinsic channel activity^5,6^. The PC-1/PC-2 complex localizes to cilia, as well as to sites of cell-cell/matrix interaction^2,3,7–9^. Although the PC-1/PC-2 complex has been described to play a role in several molecular pathways, its function remains to be understood^2^. One key activity of the polycystins receptor/channel complex has been ascribed to the capability of PC-1 to regulate key morphogenetic programs such as renal tubular morphogenesis both *in vitro*^10,11^ and *in vivo*^12,13^ as well as lymphatics development^14^. Importantly, overexpression of PC-1 was reported to drive a program of cell migration^15,16^ and front-rear polarity *via* its role in regulating focal adhesions^17^ and adherent junctions^15^ turnover as well as by regulating the actin cytoskeleton and the function of a Par3/aPKC complex^12,13,16^. The molecular mechanisms involved in this specific function of PC-1, however, remain largely elusive. Likewise, the mechanism of activation of this receptor/channel complex remains unclear. Chemical stimuli such as Extracellular Matrix (ECM) components and Wnt proteins have been shown to interact with PC-1 and to mediate its activation^18^, highlighting the possibility of this receptor being regulated by specific chemical ligands. In addition to this property, a possible role as a mechanosensor for PC-1 has been postulated on the basis of the fact that the predicted structure of PC-1 is very similar to proteins with structural and mechanical roles^19–23^. Specifically, the PC-1 extracellular region, rich in extensible Ig-like domains, shows elastic properties suggesting its role as a potential mechanosensor^22^. Furthermore, PC-1 has been shown to sense and transduce mechanical forces into biochemical signals^24–30^. In particular, based on its localization to cilia, PC-1 has been long believed to be the mechanosensor enabling renal epithelial cells to respond to the bending of cilia caused by urine flow resulting in calcium influx^24^. However, recent studies have questioned this PC-1 and PC-2 function^31^. Finally, evidence of PC-1 classification as a mechanosensor are based on its interaction with both components of the ECM and the cytoskeleton via intermediate filaments^8,32,33^. Of note, it has been shown that inactivation of integrin β1 in collecting ducts, a known positive mechanosensor of extracellular stiffness, led to inhibition of *Pkd1*-dependent cystogenesis *in vivo*^34^.

However, while the role of PC-1 extracellular domain has long been postulated to be that of a mechanical sensor, the precise signal that activates the mechanical properties of the receptor remains unknown. Polycystins are expressed in tissues other than kidney^9^, such as bone where matrix stiffness has an important impact^35^. In line with this, a recent study suggests that PC-1 has a role in regulating osteoblastogenesis and adipogenesis through interaction with the transcriptional coactivator with PDZ-binding motif (TAZ)^29,36^. Mechanical forces, such as ECM stiffness activate the PC-1/PC-2 complex leading to induction of PC-1- C-Terminal Tail (PC-1-CTT) cleavage and TAZ nuclear translocation to enhance osteoblast gene transcription and to inhibit PPARγ and adipogenesis. Supporting the role of PC-1 and TAZ in regulating bone development, Zebrafish injected with morpholinos against *Pkd1* show bone calcification defects that can be rescued by the injection of either PC-1-CTT or dominant-active TAZ mRNA^36^. Both TAZ and Yes-Associated Protein (YAP) are known as the primary downstream effectors of the Hippo pathway, which has been previously found de-regulated in ADPKD^37^, and other unrelated cystic kidney disorders^38^. More recent work has firmly established that YAP can also be activated in response to mechanical stimuli, and more precisely by extracellular stiffness generated either by the matrix^39^, or by cell density in epithelia^40^. Notably, an elegant recent study provides strong evidence that a RhoA–YAP–c-Myc axis is a direct downstream target of *PKD1* mutations^41^. Thus, *PKD1* deficiency causes constitutive activation of YAP/TAZ during kidney cystogenesis^37,41^. Here, we show that in response to the stiffness of the extracellular environment, PC-1 activates an inhibitory signal which decreases the contraction of actomyosin fibers, ultimately leading to regulation of YAP shuttling and transcriptional activity, previously shown to play an important role in renal cystogenesis^37^. Importantly, we show that PC-1 interacts in a complex of proteins regulating actomyosin contraction. Thus, we provide a direct physical and functional link between PC-1 and the inhibition of a pro-cystogenic pathway. Importantly, our data seem to suggest that the physical properties of the extracellular milieau, rather than its chemical composition, might play a key role in regulating the PC-1 receptor function, opening a new perspective on the mechanosensory activity of this receptor and the potential mechanism of pathogenesis.

## Results

### Generation of the first PC-1 interactome reveals its central role in regulation of the actomyosin machinery

**Figure 1:**
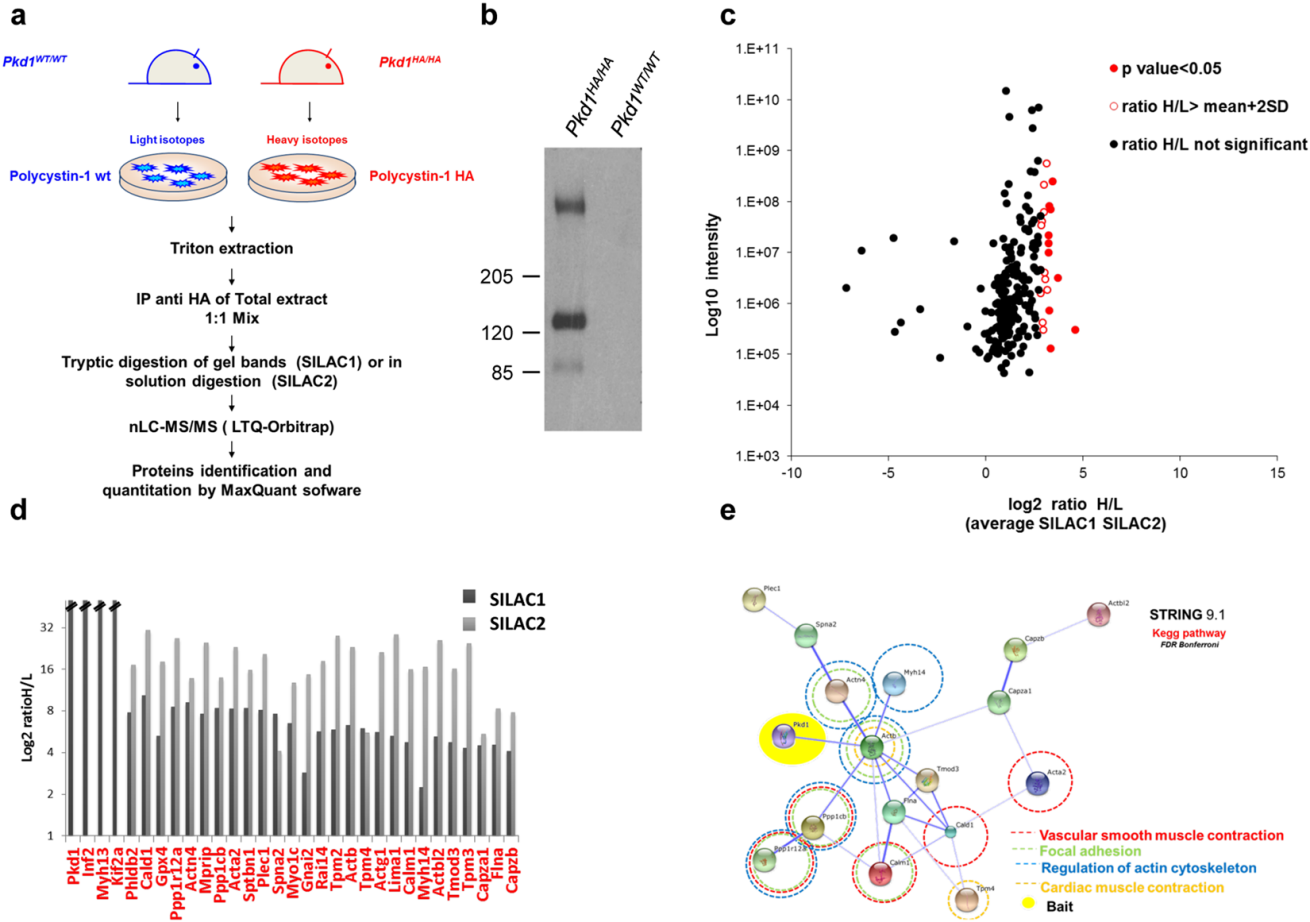
Polycystin-1 interactome study. (**a**) Proteomic workflow of the SILAC experiments for the identification of polycystin-1 interactome. (**b**) Example of HA IP showing PC-1. Full-length blot is presented in Supplementary Fig. 1. (**c**) All identified proteins were plotted according to their signal intensities and their heavy versus light ratio on logarithmic scales. (**d**) Histogram of the Log2 ratio H/L of the putative *Pkd1* interactors in SILAC1 and SILAC2. (**e**) Functional protein association networks of the putative polycystin-1 interactors as reported in STRING database. In the network, links between proteins represent interaction data supporting the network. Additionally, enriched KEGG Biological Pathways (p < 0.05) are reported and the protein associated are color code circled.

PC-1 has been proposed to play a role in multiple signaling pathways but its main physiological function is far from being understood. To gather an overview of a possible central function of PC-1, we set out to generate the interactome of the endogenous protein under stringent conditions. We isolated mouse embryonic fibroblasts (MEFs) from a *Pkd1*^*HA/HA*^ mouse model, in which an HA tag has been inserted in-frame in the last exon of the *Pkd1* gene resulting in a fully functional endogenous PC-1 protein^42^. The presence of the HA tag at the C-terminus of PC-1 allowed the immunoprecipitation (IP) of the endogenous protein using commercial beads covalently linked to an anti-HA antibody^42^. As a control, we used MEFs derived from *Pkd1*^*WT/WT*^ littermate embryos expressing equal levels of untagged PC-1. Two IP coupled to Mass Spectrometry approaches have been used, both based on SILAC (Stable Isotopes Labeling by Aminoacids in Cell culture). Briefly, *Pkd1*^*HA/HA*^ and *Pkd1*^*WT/WT*^ MEFs were grown in the presence of amino acids labeled with different isotopes (heavy-^13^C and light-^12^C, respectively) and, upon HA IP, samples were mixed and analyzed together (Fig. 1a,b). In the first approach, named SILAC1, we run the immunoprecipitated proteins on the gel and we performed in-gel proteins digestion followed by liquid chromatography mass spectrometry; in the second approach, named SILAC2, the digestion of the proteins was performed directly on the beads. While soluble proteins are better digested in solution, membrane proteins, such as PC-1, are better digested if completely denatured in the gel; therefore, a combination of the above strategies is the best choice to increase interactome coverage as much as possible. For each method two technical replicates were performed. Globally, we identified 316 proteins, 30 of them significantly enriched in the IP versus the control (Fig. 1c,d and Supplementary Table 1), including the bait itself PC-1. A scatter plot of the interactors with their heavy/light ratio is reported (Fig. 1c) together with the functional protein association networks, based on STRING database (Fig. 1e). When inspecting the list of interactors, we were pleased to see that a few known interactors were represented in the list, including the Gαi subunit of heterotrimeric G proteins, previously demonstrated to interact with PC-1^43,44^. Much to our surprise the list of interactors and the STRING analysis pointed to a single network of functional regulators of vascular smooth muscle contraction, regulation of actin cytoskeleton machinery and focal adhesions (Fig. 1e).

### PC-1 regulates actomyosin contraction

**Figure 2:**
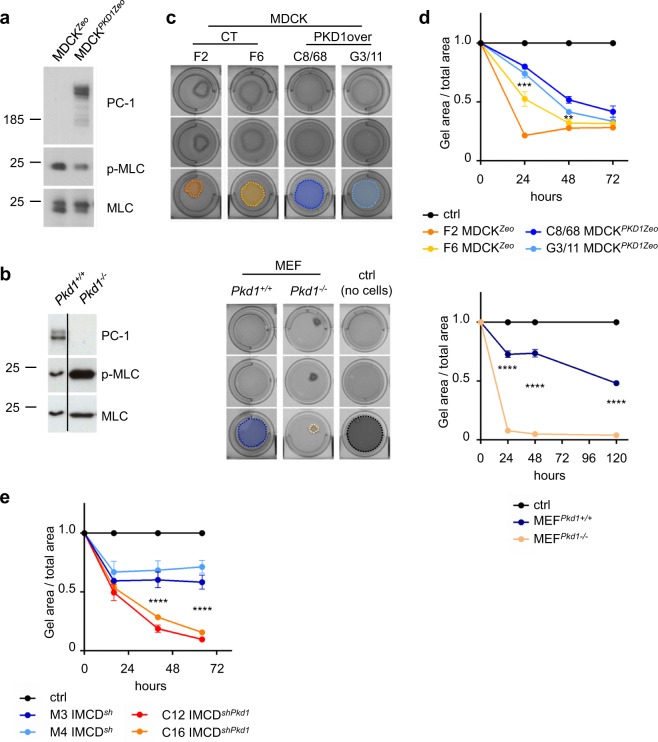
Polycystin-1 regulates phosphorylation of myosin light chain and cell contractility. (**a**) *PKD1* overexpressing epithelial MDCK cells (MDCK^*PKD1Zeo*^)^10^ display a reduced phosphorylation level of MLC in activatory sites Thr18/Ser19 compared to control cells (MDCK^*Zeo*^). Quantification of pMLC/MLC ratio in three independent experiments is presented in Supplementary Fig. 1. Bands were cropped from different parts of different gels. Full-length blots are presented in Supplementary Fig. 2. (**b**) *Pkd1*^−/−^ MEFs show hyper-phosphorylation of MLC, compared to control *Pkd1*^+/+^ MEFs. Quantification of pMLC/MLC ratio in three independent experiments is presented in Supplementary Fig. 1. Bands were cropped from different parts of different gels. Full-length blots are presented in Supplementary Fig. 2. (**c**) Top: representative pictures at 24 h of three independent experiments of collagen contraction assay (each in triplicate) revealing a lower contraction of gels with MDCK^*PKD1Zeo*^ (clones C8/68 and G3/11) cells seeded compared to those with control MDCK^*Zeo*^ (clones F2 and F6) cells. Bottom: representative pictures at 24 h of three independent experiments of collagen contraction assay (each in triplicate) revealing a higher contraction of gels with *Pkd1*^−/−^ MEFs seeded, compared to those with *Pkd1*^+/+^ MEFs. As control (ctrl), gels without seeded cells were used to determine absence of contraction. (**d**) Quantification of the contraction assay shown in c. Cells contractility is estimated as the decrease of the ratio between the gel area at specific time points and the total well area. Top: MDCK^*PKD1Zeo*^ cells (clones C8/68 and G3/11) compared to control MDCK^*Zeo*^ cells (clones F2 and F6); bottom: *Pkd1*^−/−^ MEFs compared to *Pkd1*^+/+^ MEFs. Values are mean ± SD of three replicates. Statistical analysis: one-way ANOVA, followed by Bonferroni post-test; **p < 0.01, ***p < 0.001, ****p < 0.0001. (**e**) Collagen contraction assay shows a higher contractility capacity of IMCD^*shPkd1*^ cells (clones C12 and C16) compared to control IMCD^*sh*^ cells (clones M3 and M4). Values are mean ± SD of three replicates. Statistical analysis: one-way ANOVA, followed by Bonferroni post-test; ****p < 0.0001. All experiments were performed at least three times and representative results are shown.

**Figure 3:**
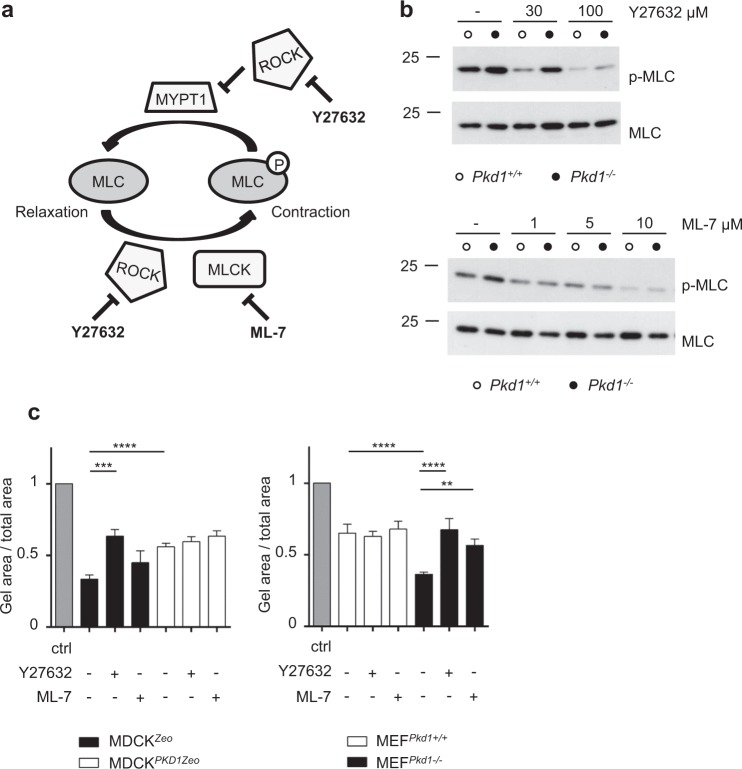
Both ROCK and MLCK inhibitors revert the effects observed in *Pkd1*^*−/−*^ cells. (**a**) Schematic representation of the complex mechanism of regulation by ROCK inhibitor Y27632 and MLCK inhibitor ML-7 on the phosphorylation status of MLC. (**b**) *Pkd1*^*−/−*^ MEFs show hyper-phosphorylation of MLC, compared to control *Pkd1*^+/+^ MEFs. Treatment with both ROCK inhibitor Y27632 100 µM (Top) and MLCK inhibitor ML-7 10 µM (bottom) is able to decrease MLC phosphorylation in *Pkd1*^*−/−*^ MEFs to the levels of control cells. Quantification of pMLC/MLC ratio in three independent experiments is presented in Supplementary Fig. 1. Bands were cropped from different parts of different gels. Full-length blots are presented in Supplementary Fig. 2. (**c**) Quantification of collagen contraction assay at 3 h showing ratio between the gel area and the total well area of MDCK^*PKD1Zeo*^ cells (left) and *Pkd1*^*−/−*^ MEFs (right). Treatment with both ROCK inhibitor Y27632 (100 µM, 4 h) and MLCK inhibitor ML-7 (10 µM, 4 h) is able to revert the cells contractility capacity to respective controls. Values are mean ± SD of three replicates. Statistical analysis: one-way ANOVA; ***p < 0.001, ****p < 0.0001. Experiments were performed at least three times and representative results are shown.

Our Mass Spectrometry data indicate that several interactors of PC-1 are proteins involved in the regulation of the actomyosin machinery. Therefore, we investigated the phosphorylation state of myosin light chain (pMLC), the main regulator of actomyosin contraction, in *Pkd1* gain and loss of function cellular models. We found that the levels of pMLC are decreased in Madine Darby Canine Kidney overexpressing PC-1 (MDCK^*PKD1Zeo*^)^10^ cells compared to controls (Fig. 2a and Supplementary Fig. 1). Conversely, MEFs isolated from *Pkd1*^*−/−*^ mice showed an increased phosphorylation state of pMLC (Fig. 2b and Supplementary Fig. 1)^45^. These results suggest that PC-1 is a negative regulator of the phosphorylation state of MLC. We therefore tested whether PC-1 can effectively regulate the contractility of cells performing a collagen contraction assay^46^. In this assay, cells are plated on top of collagen discs that are allowed to polymerize in multiwells. Collagen discs are then mechanically detached from the border of the wells, and followed over time. The contractility of cells causes a progressive reduction of the area covered by the discs, thus disc-area is a direct measure of contractility and it is inversely correlated with the capability of cells to contract. Collagen discs in the absence of cells were used as controls to define a complete absence of contraction. Consistent with their pMLC levels, MDCK^*PKD1Zeo*^ cells retracted collagen discs to a lesser extent, compared to control MDCK^*Zeo*^ cells (Fig. 2c,d). On the contrary we observed a fast and remarkable contraction capability of *Pkd1*^*−/−*^ MEFs (Fig. 2c,d) and PC-1 silenced Inner Medullary Collecting Duct (IMCD^*shPkd1*^) cells, compared to their controls (Fig. 2e). Thus, the expression levels of PC-1 inversely correlate with the pMLC levels and the capability of cells to contract. Next, we wondered which molecules upstream of the pMLC could play a role in the *Pkd1*^*−/−*^ cells. The phosphorylation levels of MLC are regulated at multiple levels, and in particular Rho kinase (ROCK) and Myosin Light Chain Kinase (MLCK)^47^ can both enhance the levels of pMLC. In addition, ROCK inhibits the phosphatase responsible for dephosphorylation of MLC (MYPT1) ultimately enhancing the contraction capability of myosin (Fig. 3a). Notably, MYPT1 appears among the direct interactors of PC-1 (PPP1r12a gene in Fig. 1d). Interestingly, both inhibitors of ROCK (Y27632) and MLCK (ML-7) were able to decrease the phosphorylation of MLC to control levels in *Pkd1*^*−/−*^ cells (Fig. 3b and Supplementary Fig. 1). In line with this, both inhibitors prevented the enhanced contraction observed in the mutant cells (Fig. 3c).

### PC-1 regulates the cellular response to extracellular stiffness

**Figure 4:**
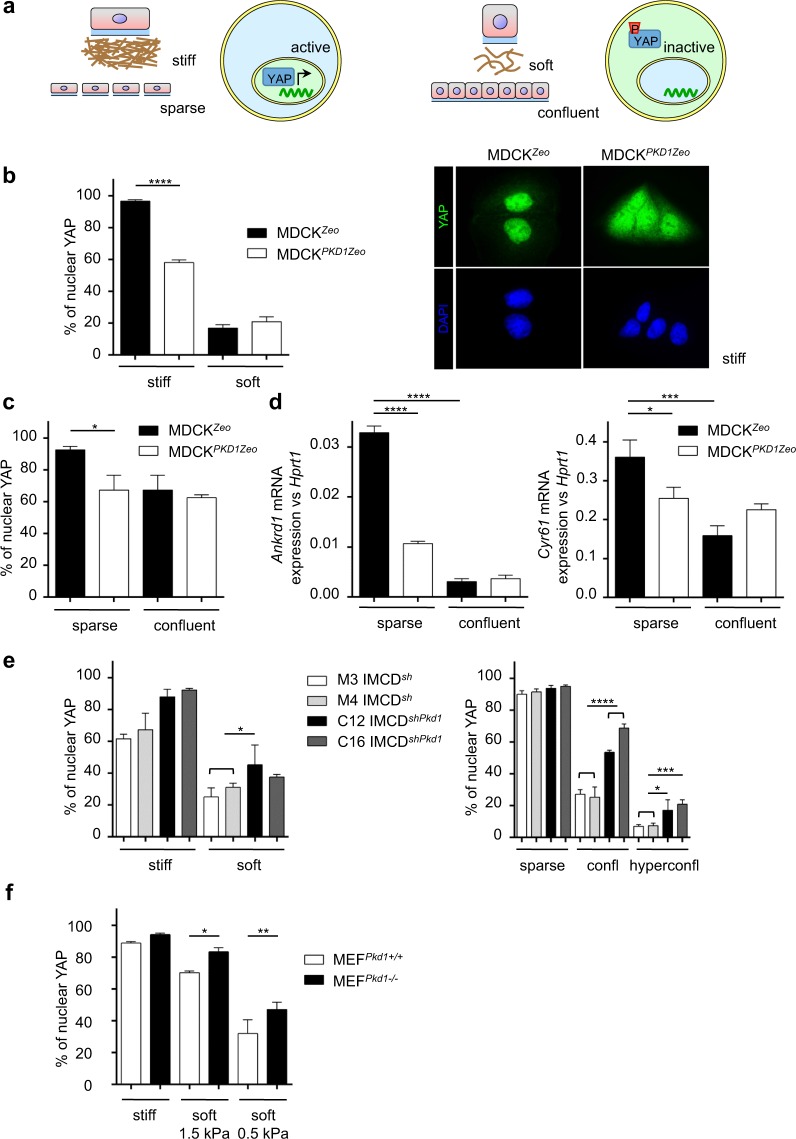
Polycystin-1-dependent myosin regulation correlates with YAP activation. (**a**) Schematic representation of YAP activation. Left: cells on stiff matrix or at low confluency show active nuclear YAP. Right: cells on soft matrix or at high confluency show inactive cytoplasmic phosphorylated YAP. (**b**) Left: percentage (%) of nuclear YAP localization quantified by immunofluorescence in MDCK^*Zeo*^ and MDCK^*PKD1Zeo*^ cells plated on collagen coated hydrogels at either stiff (40 KPa) or soft (0.5 KPa) conditions. Right: representative images of YAP localization by immunofluoescence in MDCK^*Zeo*^ and MDCK^*PKD1Zeo*^ cells in stiff condition. Values are mean + SD of three replicates (in which at least 150 cells were counted). Statistical analysis: ordinary One-way ANOVA; ****p < 0.0001. (**c**) percentage (%) of nuclear YAP localization quantified by immunofluorescence in MDCK^*Zeo*^ and MDCK^*PKD1Zeo*^ plated at either low (sparse) or high (confluent) confluency conditions. Values are mean + SD of three replicates (in which at least 150 cells were counted). Statistical analysis: ordinary One-way ANOVA; *p < 0.05. (**d**) qRT-PCR analysis for YAP targets: *Ankrd1* (left) and *Cyr61* (right) mRNA normalized towards *Hprt1* on MDCK^*PKD1Zeo*^ cells compared to control MDCK^*Zeo*^ cells plated at either low (sparse) or high (confluent) confluency conditions. Values are mean + SD of three replicates. Statistical analysis: ordinary One-way ANOVA; *p < 0.05, ***p < 0.001, ****p < 0.0001. (**e**) Left: percentage (%) of nuclear YAP localization quantified by immunofluorescence in IMCD^*shPkd1*^ cells (clones C12 and C16) compared to control IMCD^*sh*^ cells (clones M3 and M4) plated on collagen coated hydrogels at either stiff (40 KPa) or soft (0.5 KPa) conditions. Right: percentage (%) of nuclear YAP localization quantified by immunofluorescence in IMCD^*shPkd1*^ cells (clones C12 and C16) compared to control IMCD^*sh*^ cells (clones M3 and M4) plated at either low (sparse), high (confluent) or very high (hyperconfl) confluency conditions. Values are mean + SD of three replicates (in which at least 150 cells were counted). Statistical analysis: ordinary One-way ANOVA; *p < 0.05, ***p < 0.001, ****p < 0.0001. (**f**) percentage (%) of nuclear YAP localization quantified by immunofluorescence in *Pkd1*^*−/−*^ MEFs compared to control *Pkd1*^*+/+*^ MEFs plated on collagen coated hydrogels at either stiff (40 KPa) or soft (1.5 KPa and 0.5 KPa) conditions. Values are mean + SD of three replicates (in which at least 150 cells were counted). Statistical analysis: ordinary One-way ANOVA; *p < 0.05, **p < 0.01. All experiments were performed at least three times and representative results are shown.

The actomyosin pathway plays a central role in the response of cells to mechanical stimuli such as ECM elasticity or cell density, and it has been shown to regulate the activity of the transcription factor YAP in response to mechanical stress^39,48^. In particular, cells plated on stiff substrates or in sparse condition, show active nuclear localization of YAP that promotes cells proliferation and differentiation^39,49^. On the contrary in cells cultured on soft substrates or in confluent condition, YAP is retained in the cytoplasm becoming functionally inactive (Fig. 4a)^39^.

In the previous section we showed that PC-1 regulates myosin phosphorylation (Fig. 2a,b) and cell contractility (Fig. 2c,d,e). Furthermore, PC-1 structure suggests a possible role as a mechanosensor. We therefore wondered whether this receptor could be involved in sensing the rigidity of the extracellular environment. Given the central role of YAP localization in this type of response^39^, we used YAP localization as a proximal marker to measure the cellular response to extracellular stiffness in the presence or absence of PC-1.

To test this hypothesis, we plated cells on hydrogels mimicking different stiffness of the matrix with an elasticity ranging from 0.5 KPa to 40 KPa as recently reported^29,39^. Consistent with their low level of myosin phosphorylation, MDCK^*PKD1Zeo*^ cells show an attenuated response to the increased stiffness displaying higher retention of YAP in the cytoplasm, compared to control MDCK^*Zeo*^ cells (Fig. 4b). We observed a comparable response when plating MDCK^*PKD1Zeo*^ cells at low density since in epithelial cells the modulation of cell density recapitulates the response of the cell to the extracellular stiffness (Fig. 4c)^40^. As a consequence, expression of the YAP target genes *Ankrd1* and *Cyr61* is down-regulated (Fig. 4d). This is in line with PC-1 localization at the site of cell-cell and cell-matrix junction other than at the primary cilium^3,4,9,50^. According to a possible role of PC-1 in attenuating the response to extracellular stiffness, IMCD^*shPkd1*^ and *Pkd1*^*−/−*^ MEFs fail to decrease YAP activation in soft conditions compared to controls (Fig. 4e,f), in line with a recent work^29,41^. Furthermore, we tested whether in epithelial cells silencing of *Pkd1* conferred a lack of response to soft conditions generated by increased cell density^40^. Indeed, we found that IMCD^*shPkd1*^ show enhanced YAP nuclear translocation even when plated in confluent and hyper-confluent conditions (Fig. 4e).

### The dysfunctional sensitivity of extracellular stiffness results in deregulated actomyosin *in vivo*

**Figure 5:**
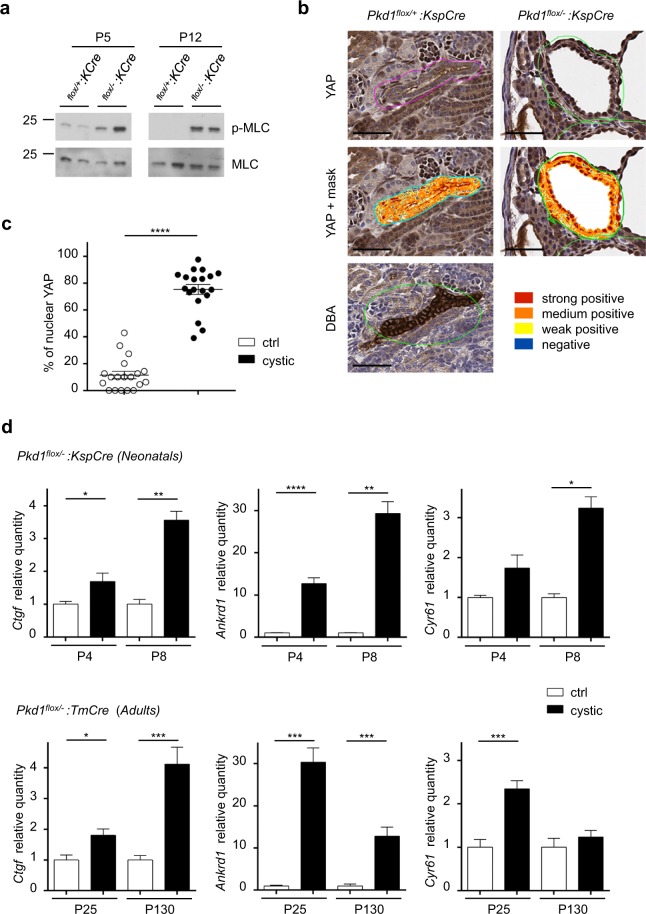
Polycystin-1 regulates phosphorylation of myosin light chain in kidneys. (**a**) Phosphorylation levels of MLC in activatory sites Thr18/Ser19 were tested by Western blot on kidney lysates. Conditional kidney inactivation of *Pkd1* gene (*Pkd1*^*flox*^^/−^:*KspCre*) causes a robust hyper-phosphorylation of MLC in mice at both P5 and P12, compared to control *Pkd1*^*flox*^^/+^:*KspCre* mice. Quantification of pMLC/MLC ratio in three independent experiments is presented in Supplementary Fig. 1. Bands were cropped from different parts of different gels. Full-length blots are presented in Supplementary Fig. 2. (**b**) IHC staining on *Pkd1*^*flox*^^/−^:*KspCre* and control *Pkd1*^*flox*^^/+^:*KspCre* kidney at P8 for YAP. Aperio image analysis software mask for the semi-automated quantification of YAP staining. IHC staining on control *Pkd1*^*flox*^^/+^: *KspCre* kidney for DBA. Scale bar = 50 μm. (**c**) Percentage (%) of nuclear YAP localization quantified as strong positive by semi-automated Aperio image analysis software on IHC slides of *Pkd1*^*flox*^^/−^: *KspCre* and control *Pkd1*^*flox*^^/+^: *KspCre* kidneys at P8 (n = 3 cystic and n = 3 littermate controls). Values are mean ± SEM of 22 cysts (224 nuclei counted for *Pkd1*^*flox*^^/+^: *KspCre*; 706 nuclei counted for *Pkd1*^*flox*^^/−^: *KspCre*). Statistical analysis: Student’s unpaired one-tailed *t*-test. ****p < 0.0001. (**d**) qRT-PCR analysis for YAP targets: *Ctgf*, *Ankrd1* and *Cyr61* mRNA normalized towards *Hprt1* on *Pkd1*^*flox/−*^: *KspCre* kidneys at P4 (n = 6 cystic and n = 6 littermate controls) and P8 (n = 3 cystic and n = 3 littermate controls) (top) and *Pkd1*^*flox/−*^: *TmCre* kidneys at P25 (n = 6 cystic and n = 6 littermate controls) and P130 (n = 8 cystic and n = 8 littermate controls) (bottom) relative to control kidneys. Values are mean + SEM. Statistical analysis: Student’s unpaired two-tailed *t*-test. *p < 0.05; **p < 0.01, ***p < 0.001, ****p < 0.0001.

**Figure 6:**
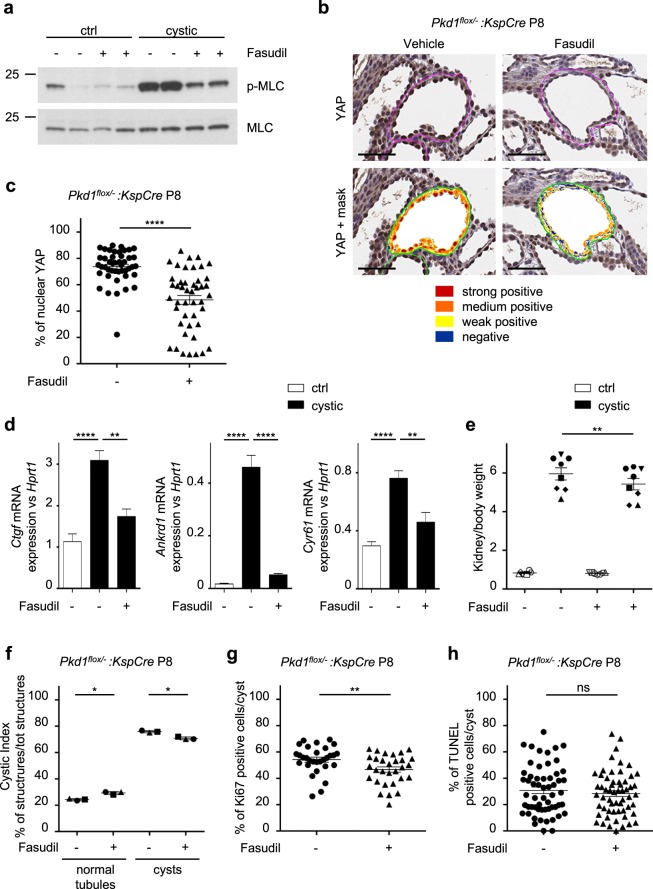
Treatment with ROCK inhibitor Fasudil amiliorates the cystic phenotype. (**a**) Phosphorylation of MLC in activatory sites Thr18/Ser19 were tested by Western blot on kidney lysates of *Pkd1*^*flox/−*^: *KspCre* mice at P8 after boost treatment with ROCK inhibitor Fasudil or vehicle. Quantification of pMLC/MLC ratio in three independent experiments is in Supplementary Fig. 1. Bands were cropped from different parts of different gels. Full-length blots are presented in Supplementary Fig. 2. (**b**) IHC staining for YAP on *Pkd1*^*flox/−*^: *KspCre* kidney at P8 after boost treatment with ROCK inhibitor Fasudil or vehicle. Aperio image analysis software mask for the semi-automated quantification of YAP staining. Scale bar = 50 μm. (**c**) Percentage (%) of nuclear YAP localization quantified as strong positive by the semi-automated Aperio image analysis software on IHC slides of *Pkd1*^*flox/−*^: *KspCre* kidneys at P8 after boost treatment with ROCK inhibitor Fasudil or vehicle (n = 3 cystic fasudil and n = 3 cystic vehicle treated mice). Values are mean ± SEM of forty five cysts (1715 nuclei counted for vehicle; 2003 nuclei counted for Fasudil). Statistical analysis: Student’s unpaired one-tailed *t*-test. ****p < 0.0001. (**d**) qRT-PCR analysis for YAP targets: *Ctgf*, *Ankrd1* and *Cyr61* mRNA normalized towards *Hprt1* on *Pkd1*^*flox/−*^: *KspCre* kidneys at P8 after boost treatment with ROCK inhibitor Fasudil or vehicle (n = 5 cystic fasudil and n = 5 cystic vehicle treated mice). Values are mean + SEM. Statistical analysis: ordinary One-way ANOVA; **p < 0.01, ****p < 0.0001 (**e**) Kidney/body weight of *Pkd1*^*flox/−*^: *KspCre* mice and *Pkd1*^*flox/+*^: *KspCre* controls after three day-treatment with ROCK inhibitor Fasudil or vehicle. Values are mean ± SEM of at least 6 animals. Statistical analysis: Student’s paired two-tailed *t*-test; **p < 0.01. (**f**) Percentage (%) of both normal tubules and cysts on total stuctures on sildes of *Pkd1*^*flox/−*^: *KspCre* kidneys at P8 after 3-day treatment with ROCK inhibitor Fasudil or vehicle (n = 3). Values are mean ± SD of at least 1000 cortical structures counted per kidney. Statistical analysis: Student’s paired one-tailed *t*-test; *p < 0.05. (**g**) Percentage (%) of Ki67 staining quantified by semi-automated Aperio image analysis software on IHC slides of *Pkd1*^*flox/−*^: *KspCre* kidneys at P8 after boost treatment with ROCK inhibitor Fasudil or vehicle (n = 3). Values are mean ± SEM of 30 cysts (1904 nuclei counted for vehicle; 1709 nuclei counted for Fasudil). Statistical analysis: Student’s unpaired one-tailed *t*-test; **p < 0.01. (**h**) Percentage (%) of apoptosis (TUNEL positive cells) quantified by immunofluorescence in slides of *Pkd1*^*flox/−*^: *KspCre* kidneys at P8 after boost treatment with ROCK inhibitor Fasudil or vehicle (n = 3 cystic fasudil and n = 3 cystic vehicle treated mice). Values are mean ± SEM of 58 cysts (2580 nuclei counted for vehicle; 2497 nuclei counted for Fasudil). Statistical analysis: Student’s unpaired one-tailed *t*-test; ns. non significant.

To address whether the role of PC-1 as a regulator of myosin activity is conserved *in vivo*, we examined pMLC levels in *Pkd1* kidney-specific conditional mutants (*Pkd1*^*flox/−*^:*KspCre*), displaying a cystic phenotype. Consistent with data in cells, we found increased phosphorylation of MLC in cystic kidneys at both P5 and P12, compared to controls (Fig. 5a and Supplementary Fig. 1). Furthermore, immunohistochemistry (IHC) analysis of kidney tissues showed the presence of nuclear YAP in the majority of epithelial cells forming cysts, whereas YAP localization was mainly cytoplasmic in non-cystic renal cells (Fig. 5b). Quantification using a semi-automated system confirmed the significant increase in cells with YAP nuclear translocation in mutant kidneys (Fig. 5c) in line with previous studies^37^. In line with this observation, we also found an enhanced expression of YAP target genes in cystic kidneys at different ages, both in a neonatal PKD model and in the adult compared to controls (Fig. 5d). Importantly, treatment of mice with the ROCK inhibitor Fasudil led to a decrease in MLC phosphorylation (Fig. 6a and Supplementary Fig. 1), YAP nuclear translocation (Fig. 6b,c) and expression of YAP target genes *Ctgf*, *Ankrd1*, *Cyr61* (Fig. 6d). These results demonstrate that in the cystic epithelia the increased YAP nuclear translocation and activity is mainly dependent on the actomyosin contraction machinery, supporting the hypothesis that cystic epithelia fail to sense and activate a proper cellular response to extracellular rigidity. Of note, treatment of mice with the inhibitor Fasudil by intra-peritoneal injection (i.p.) for three consequent days was also sufficient to ameliorate the cystic phenotype. Mice showed a reduced kidney/body weight (Fig. 6e) with a decrease of the cystic index due to the increment of normal tubules (Fig. 6f). Furthermore, analysis of proliferation by Ki67 staining showed a significant decrease in proliferation (Fig. 6g), whereas analysis of apoptosis by TUNEL did not show any difference between untreated and treated cystic kidneys (Fig. 6h). Thus, we conclude that Fasudil achieves improvement of the phenotype by reducing the proliferation rates of the cystic epithelia.

## Discussion

**Figure 7:**
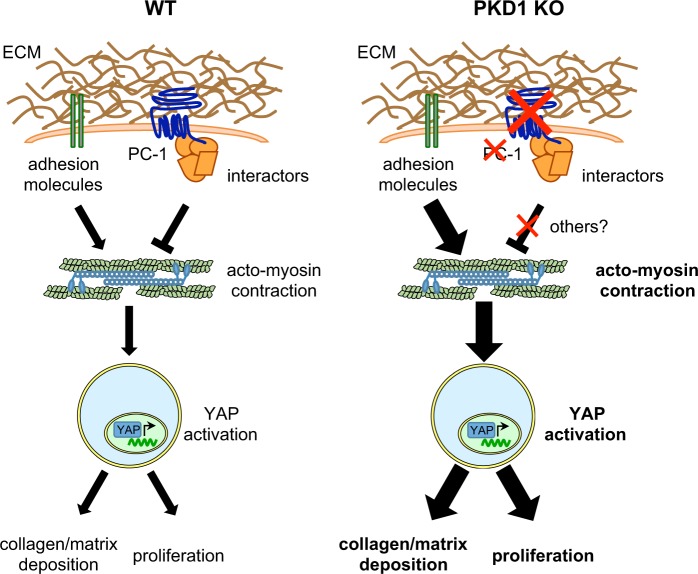
Proposed Model. Schematic representation of the proposed PC-1 role as a negative mechanosensor of extracellular stiffness. In WT condition, PC-1 acts as a negative mechanosensor of extracellular stiffness balancing the positive action of adhesion molecules like integrins. This balanced action of negative and positive mechanosensors regulates myosin phosphorylation and the consequent actomyosin contraction directly linked to YAP activation that leads to collagen/matrix deposition and cellular proliferation. In the absence of PC-1, the positive mechanosensing action of adhesion molecules is not (or less) balanced leading to increased actomyosin contraction and YAP activation, resulting in enhanced collagen/matrix deposition and cellular proliferation.

In this study, we performed a controlled screening for intracellular interactors of endogenous PC-1 and identified a new role for this receptor as a mechanosensor of the cellular response to extracellular stiffness. Of interest, PC-1 appears to act as a negative, rather than a positive, regulator of the cellular response to rigidity (Fig. 7).

The mass-spectrometry analysis of mouse embryonic fibroblasts carrying tagged endogenous PC-1 revealed that PC-1 interacts with proteins that regulate the actomyosin contraction machinery. Accordingly, we found that overexpression of PC-1 decreases pMLC levels and cellular contraction, while cells knocked-out or silenced for *Pkd1* have both effects upregulated. We further found increased pMLC in polycystic kidneys generated by kidney-specific inactivation of *Pkd1*. The actomyosin pathway plays a central role in the response of cells to extracellular rigidity. To study whether PC-1 could have a role in regulating this process we have used YAP translocation into the nucleus as a read-out of this response. Plating cells on hydrogels that mimic the different stiffness of the ECM, we have observed a reduced YAP nuclear translocation in cells overexpressing PC-1 in stiff conditions while *Pkd1* mutant cells partially failed to respond to soft condition showing an enhanced nuclear localization of YAP. Furthermore, *Pkd1* mutant kidneys display enhanced actomyosin-dependent YAP nuclear translocation and transcriptional activity. It is interesting to note here that to date only positive regulators of the extracellular response to stiffness have been identified^39^, whereas our data seem to indicate that PC-1 might be acting as a receptor for extracellular stiffness able to inhibit the cellular response via inhibition of phosphorylation of myosin. Indeed, our studies are supported by evidence that inactivation of a positive regulator of the pathway, integrin β1, results in a drastic improvement of the cystic phenotype in *Pkd1* mutant mice^34^. Our data are also in agreement with the previously reported role of PC-1 in the regulation of cell adhesion and in particular as a positive regulator of focal adhesions turnover^17,51^. It remains to be established whether PC-1 exerts this novel function via its localization at focal adhesions and cell-cell contacts or, on the contrary, whether this new function of PC-1 is also modulated by the pool of proteins present in primary cilia and future work should focus on understanding this aspect. Irrespective of the subcellular compartment in which PC-1 is active to inhibit this pathway, it is important to note here that our data might potentially explain a very interesting recent finding observed in human-derived iPS cells. Indeed, it was shown that patient derived cells carrying a *PKD1* mutation can originate a very aggressive form of cystogenesis when cultured *in vitro*, but only when the mechanical constraints are lacking, i.e. when cells are grown in suspension^52^. Based on our findings we speculate that this might be due to an inability of the cells to respond to the extracellular mechanical force given the absence of a key mechanoreceptor such as PC-1. Our data are also potentially in agreement with studies carried out in Zebrafish^53^. Indeed, silencing of *Pkd1* by morpholinos in the fish resulted in increased collagen matrix deposition and tail curvature^53^. We speculate that in this context as well, absence of PC-1 might result in a failure to sense the correct amount of collagen deposition and matrix rigidity, ultimately leading to a constitutive and excessive deposition of matrix^53^, a phenotype also observed in patient specimens^54–56^. Given the fact that deletion of integrin β1 in PKD murine models results in phenotype improvement, it would be interesting to test whether morpholinos directed against the same integrin subunit in the fish improves also this specific phenotype. Importantly, the fact that an increased matrix deposition was also observed in patient-derived tissues, points to the potential central importance of the process for the pathophysiology of ADPKD^54^.

Finally, it should be considered that, irrespectively of whether or not deregulation of actomyosin contraction is a primary event resulting in cystogenesis or, *viceversa*, it is deregulated once cysts have formed, it is important to highlight the potential of targeting this pathway as a new therapeutic approach. Indeed, we have found that the *in vivo* inhibition of the ROCK pathway using the compound Fasudil reduced YAP nuclear translocation and ameliorates PKD progression. These data are in agreement with recent work showing that the inhibitor Y27632 was able to retard disease progression in animal models^41^. While it is difficult to think that Y27632 could be used in the clinic, this provides a very important proof of principle. Our data confirm these findings and in addition show that a compound available on the market for targeting a different medical condition could offer a good opportunity for drug repurposing.

Finally, while our data do not clarify the precise mechanism by which PC-1 regulates the actomyosin contraction, they indicate that both ROCK and MLC kinase inhibitors are able to revert the pMLC levels in mutant cells. This is relevant because MLC is a Ca++ dependent kinase^47^ that might be altered primarily or secondarily in ADPKD tissues due to PC-2 dysfunction and this might concur in regulation of pMLC levels and cellular proliferation in the cystic epithelia.

In conclusion, our results indicate that applying a very stringent method for the identification of PC-1 interactors allowed the identification of few molecules pointing to a central role for PC-1 in regulation of the cellular response to the physical nature of the extracellular environment, such as the rigidity of the milieu. Even if this property is a novel central function of PC-1 potentially involved in inducing cystogenesis, it might be difficult to be corrected. However, inhibition of a downstream pathway involved in the cellular response might contribute to retard disease progression and might be explored as a potential new therapy.

## Materials and Methods

### Cell lines

MDCK cell lines stably transfected either with a full-length human *PKD1* cDNA construct (MDCK^*PKD1Zeo*^ clones C8/68, G3/11) or with an empty vector (MDCK^*Zeo*^, clones F2, F6) are described in Boletta *et al*.^10^. *Pkd1*^+/+^ (clone 11) and *Pkd1*^−/−^ (clone 14) MEFs are described in Distefano *et al*.^45^. Murine IMCD cells transduced with viral vectors expressing shRNA encoding the *Pkd1*-targeting shRNA sequence (shPkd1) (clones C12 and C16) or the scrambled sequences (shScr) (control clones M3 and M4) are already described^17^. All the cell lines were grown in 37 °C, 5% CO2 incubators, in high glucose DMEM medium (GIBCO, #41965-062), 10% FBS, 1% Penicillin-Streptomycin (GIBCO, #15070-063); for MDCK, medium was supplied with 0.5 μg/ml Geneticin (GIBCO, #11811-064) and 0.05 μg/ml Zeocin (Invitrogen, #R25001). For IMCD clones, medium was supplemented with 1 μg/ml Puromycin (Invitrogen, #A11138-03).

### Mice

The generation of both *Pkd1*^flox/*−*^:*Ksp*Cre and *Pkd1*^flox/*−*^:*Tm*Cre mice in a pure C57BL/6N genetic background has been previously described^42,57^. Intra-litter *Pkd1*^flox/+^:*Ksp*Cre and *Pkd1*^flox/+^:*Tm*Cre mice respectively were used as controls. For the *Pkd1*^flox/*−*^:*Tm*Cre model the Cre recombinase activity was induced by a single i.p. injection at P12 of Tamoxifen (Sigma-Aldrich, #T5648; 10 mg/40 g) dissolved in corn oil (Sigma-Aldrich, #C8267) by continuous shaking at 37 °C for 8 h. For all animal work, the female to male ratio was 1:1, and mice were randomized for each experiment. All animal care and all protocols used were carried out according to the Institutional regulations and specifically approved by the institutional care and use ethical committee at the San Raffaele Scientific Institute, further approved by the Italian Ministry of Health (IACUC #736).

### *In vivo* treatments

We intra-peritoneally (i.p.) injected the Rho kinase inhibitor Fasudil (LC Laboratories, #F-4660) or vehicle (NaCl) daily from P6 until P8 at 25 mg/kg of body weight. For boost treatment we i.p. injected Fasudil or vehicle (NaCl) at P8 five times every hour at 10 mg/kg of body weight.

### PC-1 immunprecipitation

*Pkd1*^*HA/HA*^ and *Pkd1*^*WT/WT*^ MEFs were grown in presence of aminoacids labeled with different isotopes (heavy-^13^C and light-^12^C, respectively). PC-1 target proteins were immunoprecipitated using anti-HA antibody. Briefly, *Pkd1*^*HA/HA*^ and *Pkd1*^*WT/WT*^ MEFs were lysate by using lysis Buffer 1% triton X-100 (Sigma-Aldrich, #1002324354). 10 mg of total extract (1 ml final volume) was incubated for 2 h with 50 μl of agarose beads at 4 °C to pre-clear the samples. The clarified supernatants were incubated overnight with anti-HA beads for the IP. Then, samples were washed 5 times with Buffer 1% triton.

Depending on the type of SILAC experiment, the heavy and light proteins immunoprecipitated were mixed 1:1 after the IP, then, for SILAC1, PC-1 IP eluates were separated by 4–12% SDS-PAGE (Life Technologies, # NP0335BOX), stained with Coomassie Brilliant Blue (Sigma-Aldrich, #B-0149) and excised in eight slices for LC-MS/MS analysis. For SILAC2, beads were resuspended in Urea 6M, Tris HCl 10 mM, proteins were digested with Lys C overnight, Urea was then diluted to 2M and trypsin digestion was performed for 3 h.

### Mass spectrometry and data analysis

Mass spectrometry analysis was performed by LC-MS/MS using an LTQ-Orbitrap mass spectrometer (ThermoScientific, Bremen, Germany). Tryptic digests were cleaned using Stage Tips as described previously^58^ and then injected in a capillary chromatographic system (EasyLC, Proxeon Biosystems, Odense, Denmark). Peptides separation occurred on a homemade column obtained with a 10-cm fused silica capillary (75 m inner diameter and 360 m outer diameter; Proxeon Biosystems) filled with Reprosil-Pur C18 3-m resin (Dr Maisch GmbH, Ammerbuch-Entringen, Germany) using a pressurized ‘packing bomb’. A gradient of eluents A [distilled water with 2% (v/v) acetonitrile, 0.1% (v/v) formic acid] and B [acetonitrile, 2% (v/v) distilled water with 0.1% (v/v) formic acid] was used to achieve separation from 8% B (at 0 min, 0.2 ml/min flow rate) to 50% B (at 80 min for SILAC1 and 180 min for SILAC2, 0.2 ml/min flow rate). The LC system was connected to the orbitrap equipped with a nanoelectrospray ion source (Proxeon Biosystems). Full-scan mass spectra were acquired in the LTQ-Orbitrap mass spectrometer in the mass range m/z 350–1500 Da and with the resolution set to 60000. The ‘lock-mass’ option was used for accurate mass measurements. The 10 most intense doubly and triply charged ions were automatically selected and fragmented in the ion trap. Target ions already selected for the MS/MS were dynamically excluded for 60 s. Protein identification and quantification were achieved using the MaxQuant software version 1.1.1.36^59^. Mass spectra were analyzed against a concatenated forward and reversed database version of uniprot_cp_mus_2011_03. The initial mass tolerance in MS mode was set to 7 ppm and MS/MS mass tolerance was 0.5 Da. Cysteine carbamidomethylation was searched as a fixed modification, whereas N-acetyl protein and oxidized methionine were searched as variable modifications. Labeled arginine and lysine were also specified as variable modifications. SILAC peptides and proteins quantification were performed automatically with MaxQuant using default settings as parameters. Peptides and proteins were accepted with a false-discovery rate of 0.01, minimum one unique peptide identified per protein. To identify PC-1 interactors, we analyzed the Gaussian distribution and we used the standard deviation of the sample as a cut-off to identify outliers. Outliers were selected based on p < 0.05 as the most stringent criteria and a cutoff ratio H/L > mean + 2 SD considering the Gaussian distribution.

### Antibodies and inhibitors

For Western blot analysis we used the following antibodies: PC-1 LRR (Santa Cruz Biotechnology, #sc-130554; 1:1000), HA High Affinity (Roche, #11867423001; 1:1000) p-MLC^Thr18/Ser19^ (Cell Signaling, #3674S; 1:1000), MLC (Cell Signaling, #3672; 1:1000). HRP-conjugated secondary antibodies were from GE Healthcare, anti-rabbit IgG HRP linked (#934V) anti-mouse IgG HRP linked (#NA9310V) and anti-rat IgG HRP linked (#NA935V). For IF and IHC staining we used YAP (Santa Cruz Biotechnology, #sc-101199; 1:200 or Cell Signaling, #4912; 1:50 respectively), Ki67 (Thermo Fisher Scientific, #RM-9106; 1:100) antibodies and DAPI (Santa Cruz Biotechnology, #sc-3598; 1:5000). Alexa-488 secondary anti mouse antibody were from Invitrogen (#A11001). For IP we used anti-HA affinity matrix (Roche, #11815016001). Rho kinase inhibitor Y27632 and MLCK inhibitor ML-7 were from Tocris (#1254 and #4310, respectively).

### Immunohistochemistry

For histological analysis mice were sacrificed at the indicated time, kidneys were collected, weighted and fixed in formalin 10% (BioOptica, #05-01005Q), included in paraffin and cut 5 μm/slides. Kidney sections were air-dried and rehydrated in PBS (Sigma-Aldrich, #P4417). For YAP staining kidney sections were incubated with YAP antibody and 5 min in Eosin G (BioOptica, #05-10002/L); For Ki67 staining kidney sections were incubated with Ki67 antibody and 2 min in Hematoxylin (BioOptica, #05-06015/L). Sections were then washed, processed through a dehydration alcohol scale and mounted in DPX (Sigma-Aldrich, #06522). For the semi-automated quantification, slides were acquired with Aperio AT2 digital scanner at magnification of 40 × (Leica Biosystems) and analyzed with Imagescope (Leica Biosystem). YAP and Ki67 nuclear staining per cyst was performed by Aperio Image analysis software (Leica Biosystems) and counted manually.

### Cystic index

To quantify the Cystic Index we manually counted at least 1000 tubular structures in cortical sections of kidneys stained with Hematoxylin-Eosin. To quantify the rate of cystic kidneys we applied a grid of squares 13.625 μm large to sections of kidneys stained with Hematoxylin-Eosin. We marked each cross with a dot and we manually counted the number of dots inside the lumen on three litters. We determined the degree of dilatation according to 1 dot: normal tubules; >2 dots: dilated tubules and cysts as in^60^.

### Immunofluorescence

For immunofluorescence staining, cells seeded on a glass or on a hydrogels-covered glass were washed in PBS, fixed 10 min in 4% Paraformaldehyde and permeabilized in 0.2% Triton X-100 in PBS. After 1 h blocking in 3% BSA (Sigma-Aldrich, #A7906) in PBS at RT, cells were incubated over night at 4 °C with primary antibody diluted in 3% BSA in PBS. Cells were then incubated with secondary antibody diluted in 3% BSA in PBS for 1 h at RT and nuclei were stained with DAPI. Glasses were then mounted with Fluorescence Mounting medium (Dako, #S3023). For the apoptotic assay, kidney sections were analyzed by the DeadEnd Flurometric transferase-mediated dUTP nick-end labeling (TUNEL) system kit (Promega, #G3250). Images were obtained using Zeiss Axio Observer.Z1 microscope. Quantification of both YAP and TUNEL nuclear staining was performed manually.

### Real-time pcr analysis

Total RNA was isolated from cells or kidneys using the RNAspin Mini kit (GE Healthcare, #25-0500-72). cDNA was obtained by reverse transcription of extracted RNA using Oligo(dt)_15_ primers (Promega, #C1101) and ImProm-II Reverse Transcriptase (Promega, #A3802). Quantitative Real Time PCR analysis was performed on technical duplicates using SYBR Green I master mix (Roche, #04887352001) on LightCycler 480 Instrument (Roche). Primer sequences for qRT-PCR:

*mHprt1* fw 5′-TTATGTCCCCCGTTGACTGA-3′

*mHprt1* rev 3′-ACATTGTGGCCCTCTGTGTG-5′

*mCtgf* fw 5′-GCTTGGCGATTTTAGGTGTC-3′

*mCtgf* rev 3′-CAGACTGGAGAAGCAGAGCC-5′

*mAnkrd1* fw 5′-CAGTGCAACACCAGATCCAT-3′

*mAnkrd1* rev 3′-ATGCCAAGGACAGAGAAGGA-5′

*mCyr61* fw 5′-TTTACAGTTGGGCTGGAAGC-3′

*mCyr61*rev 3′-AAGGGGTTGGAATGCAATTT-5′

*Canine Hprt1* fw 5′-ACACTGGGAAAACAATGCAGAC-3′

*Canine Hprt1* rev 3′-TCAGGTTTATAGCCAACACTTCG-5′

*Canine Ankrd1* fw 5′-TGGAGCCCAGATCGAATTCC-3′

*Canine Ankrd1*rev 3′-CACTGAGTCATGCAGAGGGG-5′

*Canine Cyr61* fw 5′-TCTGTGACGACGATGATGCC-3′

*Canine Cyr61* rev 3′-GTACAGGATTCGAGGCTCCG-5′

### Western blot analysis

For Western blot analysis cells or kidneys were lysed in lysis buffer solution of 150 mM NaCl (Sigma-Aldrich, #s9625), 20 mM Na_2_HPO_4_ (BDH, #10494L)/NaH_2_PO_4_ (BDH, #102455S), 10% Glycerol (Sigma-Aldrich, #G7757), 1% Triton X-100 (pH 7.2), complete protease inhibitor cocktail (Roche, #11836145001) and phosphatase inhibitors [1 mM final concentration of glycerophosphate (Sigma-Aldrich, #G9891), sodium orthovanadate (Sigma-Aldrich, #S6508) and sodium fluoride (Sigma-Aldrich, #S6521)]. Total lysates were then quantified with Bio-Rad Protein Assay Dye reagent (Bio-Rad, #500–0006) and Laemmli buffer at a final concentration 2x was added to the samples. Proteins were next resolved in 4–12% Tris-Glycine gradient gels (Life Technologies, # NP0335BOX) and then transferred onto Immobilon-P polyvinylidene fluoride (PVDF) membranes (Millipore, #IPVH00010). We then blocked membranes with 5% milk in Tris-buffered saline, Tween 20 (Sigma-Aldrich, #P1379) (TBS-T). All the primary antibodies for Western blot analysis were diluted in 3% BSA in TBS-T. HRP-conjugated secondary antibodies were diluted 1:10000 in 5% milk, TBS-T and detection was made with ECL (GE Healthcare, #RPN2106) alone or supplied with 10% SuperSignal West Femto (Thermo Fisher Scientific, #34095) when necessary.

### Hydrogels of polyacrylamide

In order to test the cellular response to the mechanical properties of the extra-cellular matrix we have prepared hydrogels of polyacrylamide with modulable rigidity. The protocol described by Tse *et al*.^61^ has been adapted as follows: 15 mm of diameter round cover glasses were incubated with 250 μl 0.1M NaOH (Riedel de Haen, #30620) and placed at 80 °C until the liquid has evaporated. Glasses were then incubated with 125 μl of 3-aminopropyltriethoxysilane (APES) (Thermoscience, #80370) for 5 min and then placed into a multi-well plate and washed with ddH2O three times for 5 min. Glasses were then incubated with 250 μl of 0.5% glutaraldehyde (Sigma-Aldrich, #G6257) in PBS for 30 min. After removing the glutaraldehyde and allow glasses to dry, we treated the glass surface of slide glasses with 100 μl of Diclorodimethylsilane (DCDMS) (Merck, #8034520250) for 5 min and then washed with ddH2O. We prepared acrylamide/bis-acrylamide (Sigma-Aldrich, #A2792) solutions at different proportions according to the desired rigidity and added 10% APS (1:100) and TEMED (1:1000) (BDH, #4430836) quickly before pipetting 10 μl of solution onto the slide glass. We put the cover glasses with the side treated towards the drop and let the gel polymerized for 10 min. We then washed the obtained hydrogels with ddH2O and store them at 4 °C in PBS. The day before plating cells, we treated the hydrogels with 500 μl of sulfo-SANPAH (0.2 mg/ml sulfosuccinimidyl-6-(4’-azido-2’-nitrophenylamino-hexanoate) (Thermo Fisher Scientific, #22589) under UV (365 nm) for 10 min. We washed three times with HEPES (Sigma-Aldrich #H3375) 50 mM pH 8.5 and coated the hydrogels with 50 μl of Collagen type 1 Rat tail solution (Corning, #354236) in HEPES 0.10 mg/ml at 37 °C O/N. The following day, we transfered the hydrogels into a 12-well plates and washed them three times with PBS. After treating the hydrogels for 30 min with UV, we seeded 35000 cells/hydrogels.

### Contraction assay

To monitor cellular contractility *in vitro*, cells were plated on thin collagen gels. Cellular contraction caused a decrease in the collagen area that can be quantified to estimate cells contractility during time^46^. The day before plating the cells we coated a 12-well plate with 500 μl/well of a Collagen solution in PBS at 37 °C O/N: the solution consist in 7 Volumes of Collagen Type 1 (1 mg/ml), 2 Volumes of DMEM (GIBCO, #52100-039) and 1 Volume of Neutralizing Buffer (2.2 gr NaHCO3 (Carlo Erba Reagents, #478537), 4.77 gr HEPES, NaOH 0.05M in a final volume of 100 ml), FBS 5%. The day after, we seeded 150000 cells/well and waited the cells to adhere for about 4 h. We then detached the edges of the gel form the well and monitor the changes in the gel area taking pictures over time. The analysis of the images of the gel area/well area were performed with the ImageJ software.

### Statistical analysis

Differences between averages were established with Student’s *t-*test or one way ANOVA analysis of variance as indicated in the figure legends; Bonferroni’s post-test was carried out for multiple comparisons.

## Supplementary information

Supplementary file and figures

Supplementary data

## References

[1] Torres VE, Harris PC, Pirson Y (2007). Autosomal dominant polycystic kidney disease. Lancet.

[2] Ong, A. C. & Harris, P. C. A polycystin-centric view of cyst formation and disease: the polycystins revisited. *Kidney Int* (2015). 10.1038/ki.2015.207 PMC4589452 26200945

[3] Boletta A, Germino GG (2003). Role of polycystins in renal tubulogenesis. Trends Cell Biol.

[4] Chapin HC, Caplan MJ (2010). The cell biology of polycystic kidney disease. J Cell Biol.

[5] Hanaoka K (2000). Co-assembly of polycystin-1 and -2 produces unique cation-permeable currents. Nature.

[6] Parnell SC (2002). Polycystin-1 activation of c-Jun N-terminal kinase and AP-1 is mediated by heterotrimeric G proteins. J Biol Chem.

[7] Yoder BK, Hou X, Guay-Woodford LM (2002). The polycystic kidney disease proteins, polycystin-1, polycystin-2, polaris, and cystin, are co-localized in renal cilia. J Am Soc Nephrol.

[8] Ibraghimov-Beskrovnaya O (2000). Strong homophilic interactions of the Ig-like domains of polycystin-1, the protein product of an autosomal dominant polycystic kidney disease gene, PKD1. Hum Mol Genet.

[9] Peters DJ (1999). Cellular localization and tissue distribution of polycystin-1. J Pathol.

[10] Boletta A (2000). Polycystin-1, the gene product of PKD1, induces resistance to apoptosis and spontaneous tubulogenesis in MDCK cells. Mol Cell.

[11] Rowe I, Chiaravalli M, Piontek KB, Germino GG, Boletta A (2014). Impaired glomerulogenesis and endothelial cell migration in Pkd1-deficient renal organ cultures. Biochem Biophys Res Commun.

[12] Castelli M (2013). Polycystin-1 binds Par3/aPKC and controls convergent extension during renal tubular morphogenesis. Nat Commun.

[13] Nigro EA, Castelli M, Boletta A (2015). Role of the Polycystins in Cell Migration, Polarity, and Tissue Morphogenesis. Cells.

[14] Outeda P (2014). Polycystin signaling is required for directed endothelial cell migration and lymphatic development. Cell Rep.

[15] Boca M (2007). Polycystin-1 induces cell migration by regulating phosphatidylinositol 3-kinase-dependent cytoskeletal rearrangements and GSK3beta-dependent cell cell mechanical adhesion. Mol Biol Cell.

[16] Yao G (2014). Polycystin-1 regulates actin cytoskeleton organization and directional cell migration through a novel PC1-Pacsin 2-N-Wasp complex. Hum Mol Genet.

[17] Castelli M (2015). Regulation of the microtubular cytoskeleton by Polycystin-1 favors focal adhesions turnover to modulate cell adhesion and migration. BMC Cell Biol.

[18] Kim E (1999). The polycystic kidney disease 1 gene product modulates Wnt signaling. J Biol Chem.

[19] Boca M (2006). Polycystin-1 induces resistance to apoptosis through the phosphatidylinositol 3-kinase/Akt signaling pathway. J Am Soc Nephrol.

[20] Forman JR, Qamar S, Paci E, Sandford RN, Clarke J (2005). The remarkable mechanical strength of polycystin-1 supports a direct role in mechanotransduction. J Mol Biol.

[21] Bhoonderowa L (2016). Polycystins and intercellular mechanotransduction: A precise dosage of polycystin 2 is necessary for alpha-actinin reinforcement of junctions upon mechanical stimulation. Exp Cell Res.

[22] Qian F, Wei W, Germino G, Oberhauser A (2005). The nanomechanics of polycystin-1 extracellular region. J Biol Chem.

[23] Xu M (2013). Analysis of the REJ Module of Polycystin-1 Using Molecular Modeling and Force-Spectroscopy Techniques. J Biophys.

[24] Nauli SM (2003). Polycystins 1 and 2 mediate mechanosensation in the primary cilium of kidney cells. Nat Genet.

[25] Chauvet V (2004). Mechanical stimuli induce cleavage and nuclear translocation of the polycystin-1 C terminus. J Clin Invest.

[26] Pedrozo Z (2015). Polycystin-1 Is a Cardiomyocyte Mechanosensor That Governs L-Type Ca2+ Channel Protein Stability. Circulation.

[27] Sharif-Naeini R (2009). Polycystin-1 and -2 dosage regulates pressure sensing. Cell.

[28] Peyronnet R (2013). Piezo1-dependent stretch-activated channels are inhibited by Polycystin-2 in renal tubular epithelial cells. EMBO Rep.

[29] Xiao Z (2018). Polycystin-1 interacts with TAZ to stimulate osteoblastogenesis and inhibit adipogenesis. J Clin Invest.

[30] Gargalionis, A. N., Basdra, E. K. & Papavassiliou, A. G. Polycystins in disease mechanobiology. *J Cell Biochem* (2018). 10.1002/jcb.28127 30461048

[31] Delling M (2016). Primary cilia are not calcium-responsive mechanosensors. Nature.

[32] Streets AJ (2003). Functional analysis of PKD1 transgenic lines reveals a direct role for polycystin-1 in mediating cell-cell adhesion. J Am Soc Nephrol.

[33] Xu GM (2001). Polycystin-1 interacts with intermediate filaments. J Biol Chem.

[34] Lee K, Boctor S, Barisoni LM, Gusella GL (2015). Inactivation of integrin-beta1 prevents the development of polycystic kidney disease after the loss of polycystin-1. J Am Soc Nephrol.

[35] Chauvet V (2002). Expression of PKD1 and PKD2 transcripts and proteins in human embryo and during normal kidney development. Am J Pathol.

[36] Merrick D (2019). Polycystin-1 regulates bone development through an interaction with the transcriptional coactivator TAZ. Hum Mol Genet.

[37] Happe H (2011). Altered Hippo signalling in polycystic kidney disease. J Pathol.

[38] Grampa V (2016). Novel NEK8 Mutations Cause Severe Syndromic Renal Cystic Dysplasia through YAP Dysregulation. PLoS Genet.

[39] Dupont S (2011). Role of YAP/TAZ in mechanotransduction. Nature.

[40] Aragona M (2013). A mechanical checkpoint controls multicellular growth through YAP/TAZ regulation by actin-processing factors. Cell.

[41] Cai J (2018). A RhoA-YAP-c-Myc signaling axis promotes the development of polycystic kidney disease. Genes Dev.

[42] Wodarczyk C (2009). A novel mouse model reveals that polycystin-1 deficiency in ependyma and choroid plexus results in dysfunctional cilia and hydrocephalus. PLoS One.

[43] Parnell SC (1998). The polycystic kidney disease-1 protein, polycystin-1, binds and activates heterotrimeric G-proteins *in vitro*. Biochem Biophys Res Commun.

[44] Parnell SC (2018). A mutation affecting polycystin-1 mediated heterotrimeric G-protein signaling causes PKD. Hum Mol Genet.

[45] Distefano G (2009). Polycystin-1 regulates extracellular signal-regulated kinase-dependent phosphorylation of tuberin to control cell size through mTOR and its downstream effectors S6K and 4EBP1. Mol Cell Biol.

[46] Pins GD, Collins-Pavao ME, Van De Water L, Yarmush ML, Morgan JR (2000). Plasmin triggers rapid contraction and degradation of fibroblast-populated collagen lattices. J Invest Dermatol.

[47] Shen Q, Rigor RR, Pivetti CD, Wu MH, Yuan SY (2010). Myosin light chain kinase in microvascular endothelial barrier function. Cardiovasc Res.

[48] Shah JS (2018). Biomechanics and mechanical signaling in the ovary: a systematic review. J Assist Reprod Genet.

[49] Xu D (2018). Scribble influences cyst formation in autosomal-dominant polycystic kidney disease by regulating Hippo signaling pathway. FASEB J.

[50] Boletta A (2001). Biochemical characterization of bona fide polycystin-1 *in vitro* and *in vivo*. Am J Kidney Dis.

[51] Wilson PD, Geng L, Li X (1999). & Burrow, C.R. The PKD1 gene product, “polycystin-1,” is a tyrosine-phosphorylated protein that colocalizes with alpha2beta1-integrin in focal clusters in adherent renal epithelia. Lab Invest.

[52] Cruz NM (2017). Organoid cystogenesis reveals a critical role of microenvironment in human polycystic kidney disease. Nat Mater.

[53] Mangos S (2010). The ADPKD genes pkd1a/b and pkd2 regulate extracellular matrix formation. Dis Model Mech.

[54] Carone FA, Butkowski RJ, Nakamura S, Polenakovic M, Kanwar YS (1994). Tubular basement membrane changes during induction and regression of drug-induced polycystic kidney disease. Kidney Int.

[55] Wilson PD, Norman JT, Kuo NT, Burrow CR (1996). Abnormalities in extracellular matrix regulation in autosomal dominant polycystic kidney disease. Contrib Nephrol.

[56] Carone FA, Bacallao R, Kanwar Y (1998). Role of the matrix in autosomal dominant polycystic kidney disease. Ren Fail.

[57] Chiaravalli M (2016). 2-Deoxy-d-Glucose Ameliorates PKD Progression. J Am Soc Nephrol.

[58] Rappsilber J, Mann M, Ishihama Y (2007). Protocol for micro-purification, enrichment, pre-fractionation and storage of peptides for proteomics using StageTips. Nat Protoc.

[59] Cox J, Mann M (2008). MaxQuant enables high peptide identification rates, individualized p.p.b.-range mass accuracies and proteome-wide protein quantification. Nat Biotechnol.

[60] Rowe I (2013). Defective glucose metabolism in polycystic kidney disease identifies a new therapeutic strategy. Nat Med.

[61] Tse, J. R. & Engler, A. J. Preparation of hydrogel substrates with tunable mechanical properties. *Curr Protoc Cell Biol* Chapter 10, Unit 10 16 (2010). 10.1002/0471143030.cb1016s47 20521229

