# Supplementary material for: Polycystin-1 Regulates Actomyosin Contraction and the Cellular Response to Extracellular Stiffness
Source: Sci Rep. 2019 Nov 12;9:16640. doi: 10.1038/s41598-019-53061-0 (PMC6851149; doi:10.1038/s41598-019-53061-0)
Supplement: Supplementary file 1 — Supplementary file and figures [file 41598_2019_53061_MOESM1_ESM.pdf]

Polycystin-1 Regulates Actomyosin Contraction and the Cellular Response to Extracellular Stiffness

Elisa A. Nigro[1], Gianfranco Distefano[1], Marco Chiaravalli[1], Vittoria Matafora[2], Maddalena Castelli[1], Angela Pesenti Gritti[1], Angela Bachi[2] and Alessandra Boletta[1*]

1. IRCCS San Raffaele Scientific Institute, Molecular Basis of Cystic Kidney Disorders, Division of Genetics and Cell Biology, Milan, Italy
2. IFOM-FIRC Institute of Molecular Oncology, Milan, Italy

•***Corresponding Author:** Alessandra Boletta, Via Olgettina 58, 20132 Milano, Italy. E-mail: boletta.alessandra@hsr.it

Supplementary Table 1: **Polycystin-1 interactome study.**

Upon Polycystin-1 pull down, interactors were identified by SILAC-Mass Spectrometry analysis. A list of the PC-1 interactors is reported. For each protein, SILAC heavy/light ($Pkd1^{HA/HA}$/$Pkd1^{WT/WT}$) chain ratios and the relative intensity values for both biological replicates are reported in the table. Notably, Polycystin-1 and several other proteins are not identified in the light form, therefore those proteins are not presenting any heavy/light ratio.

Supplementary Figure 1: Western Blots quantifications.

Quantification of three distinct western blot analysis, showing ratio of expression between pMLC/MLC for all the blots included in the manuscript. Values are mean ± SD of three independent experiments . Statistical analysis: Student's paired one-tailed *t*-test. *p < 0.05, **p < 0.01 ***p < 0.001.

Supplementary Figure 2: Full-lenght Western Blots.

Uncropped images of all the blots included in the manuscript.

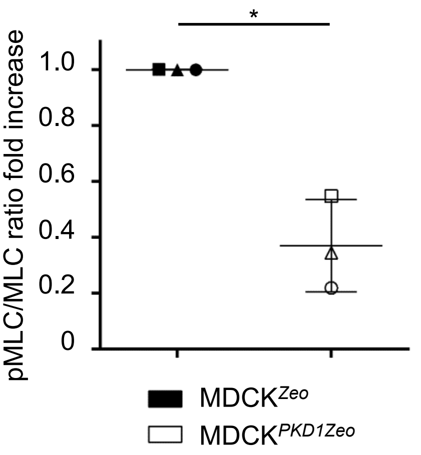

Quantification of Figure 2A

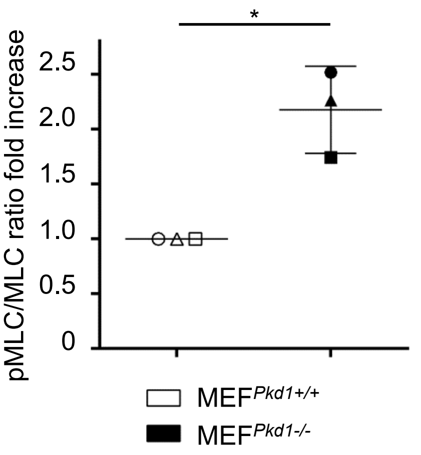

Quantification of Figure 2B

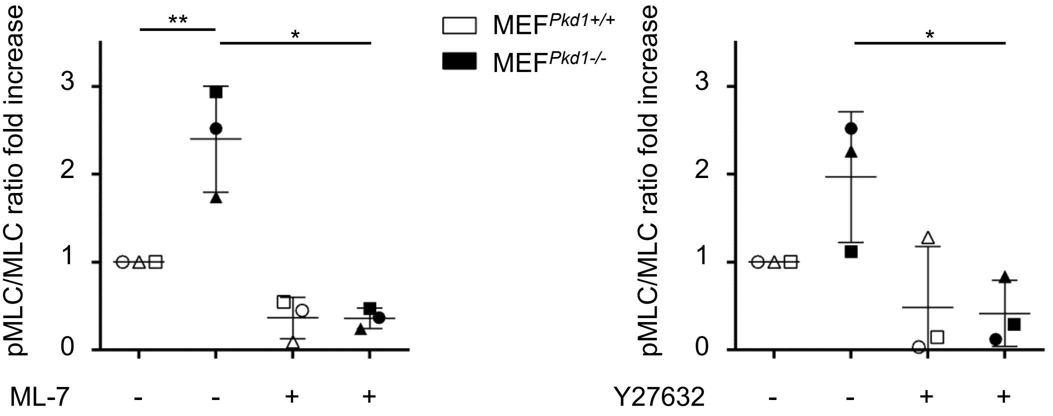

Quantification of Figure 3B

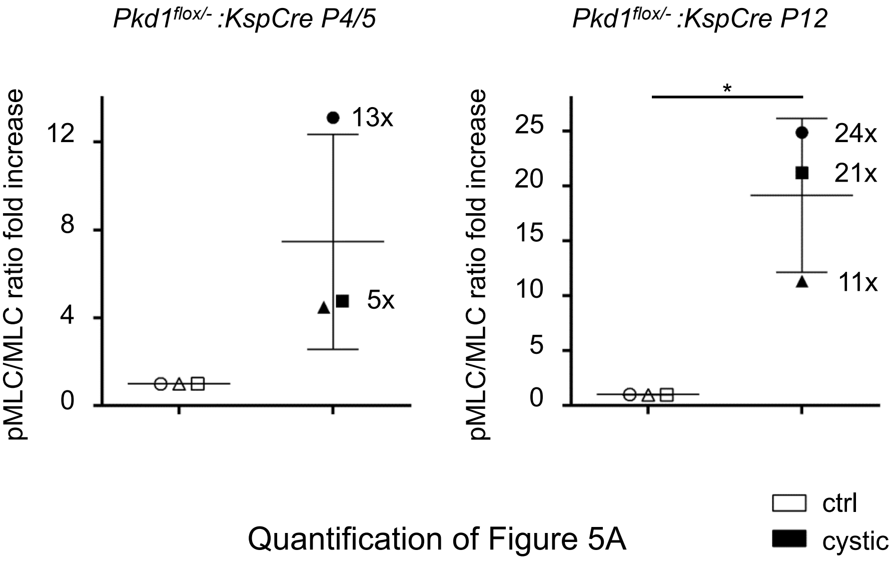

Quantification of Figure 5A

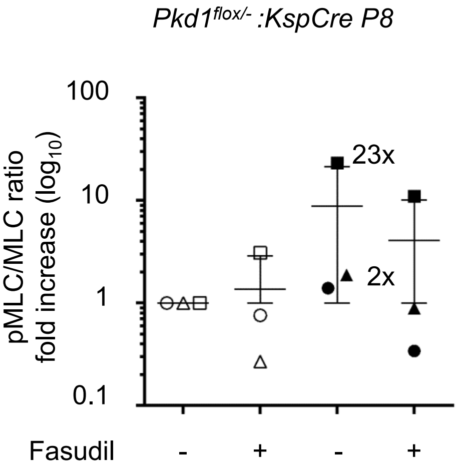

Quantification of Figure 6A

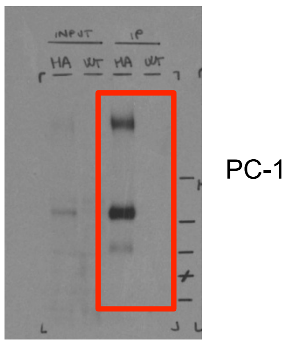

Uncropped image of blot presented in Figure 1B

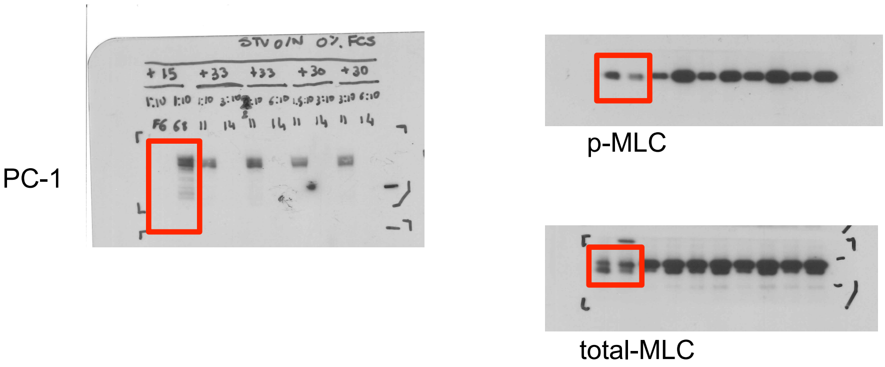

Uncropped images of blots presented in Figure 2A

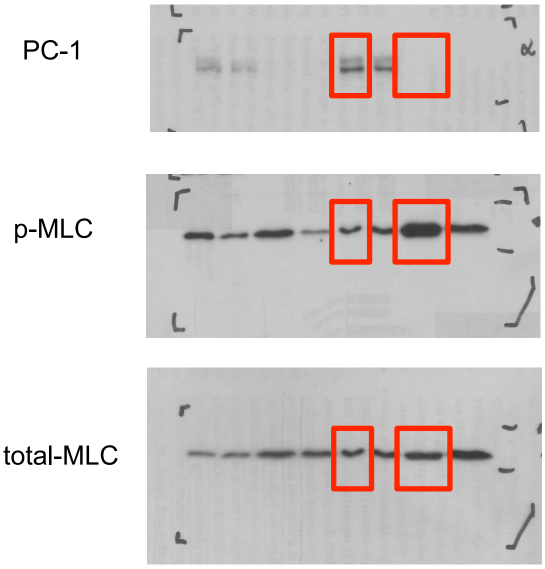

Uncropped images of blots presented in Figure 2B

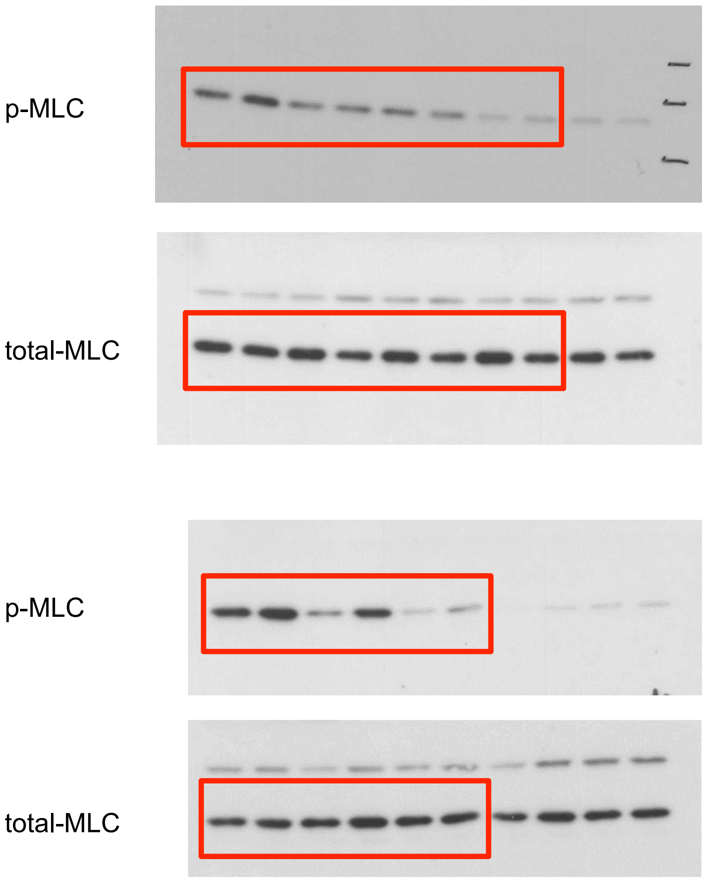

Uncropped images of blots presented in Figure 3B

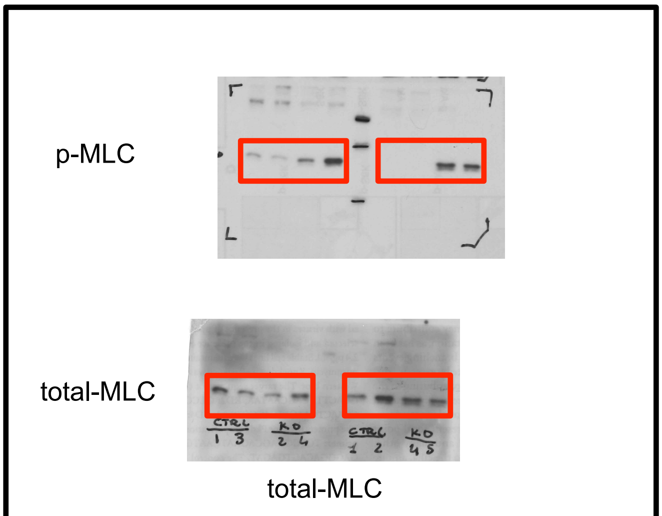

Uncropped images of blots presented in Figure 5A

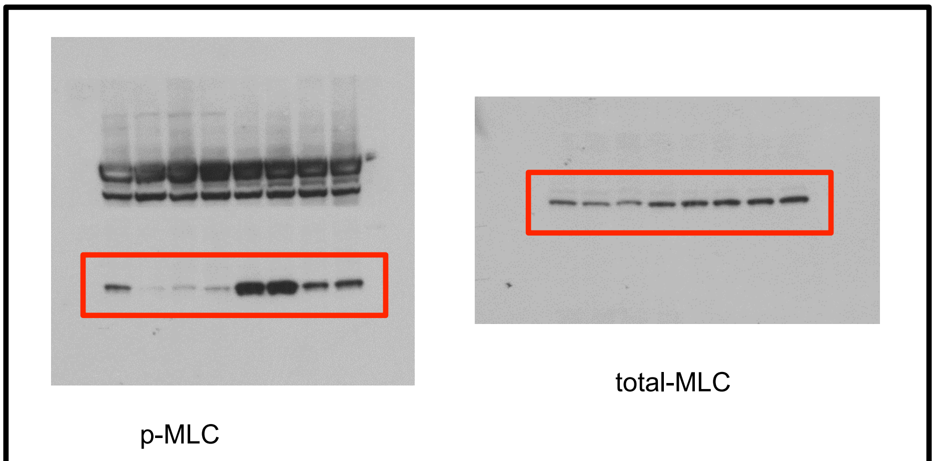

Uncropped images of blots presented in Figure 6A

Nigro et al. Supp Fig. 2
